# Supplementary material for: Automated selection of mid-height intervertebral disc slice in traverse lumbar spine MRI using a combination of deep learning feature and machine learning classifier
Source: PLoS One. 2022 Jan 13;17(1):e0261659. doi: 10.1371/journal.pone.0261659 (PMC8758114; doi:10.1371/journal.pone.0261659)

Dear readers,

Thank you for your interest in our paper and work. In this document, you can find the information on how to access the data used in our research as well as the source code (MATLAB and PYTHON) that we used when implementing the methodology.

The dataset and source code are put in one zip file called Source and Dataset.zip and the file is hosted in Mendeley Data. The URL to the data is below (please copy and paste the address to your Web Browser instead of clicking it).

https://data.mendeley.com/datasets/ggjtzh452d/1

You will need to have an Elsevier account to access this data to comply with the term and condition of the host. But, creating one is straightforward and free. We provide a short instruction on how to create one in this document.

If you have any questions please feel free to contact me at [s.sudirman@ljmu.ac.uk](mailto:s.sudirman@ljmu.ac.uk)

Kind regards

Sud Sudirman

**Instructions on how to Access the Data**

Please go to https://data.mendeley.com/datasets/ggjtzh452d/1 to download the dataset.

You will be prompted to log in to Elsevier on the page. If you already have an Elsevier account you should be directed to the dataset page after you log in. If not, you can register a free account. Just type in your email address in the box provided and click Continue.


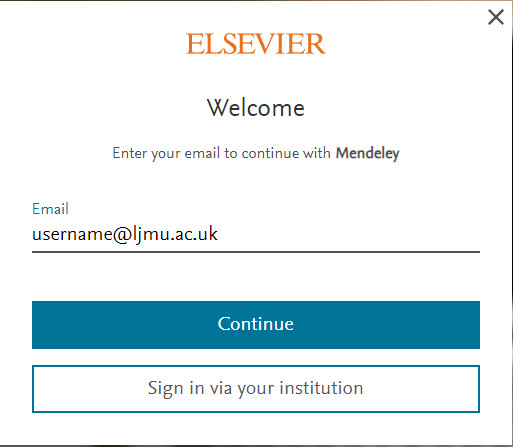


You will then be prompted for your name and a preferred password as shown in the figure below.


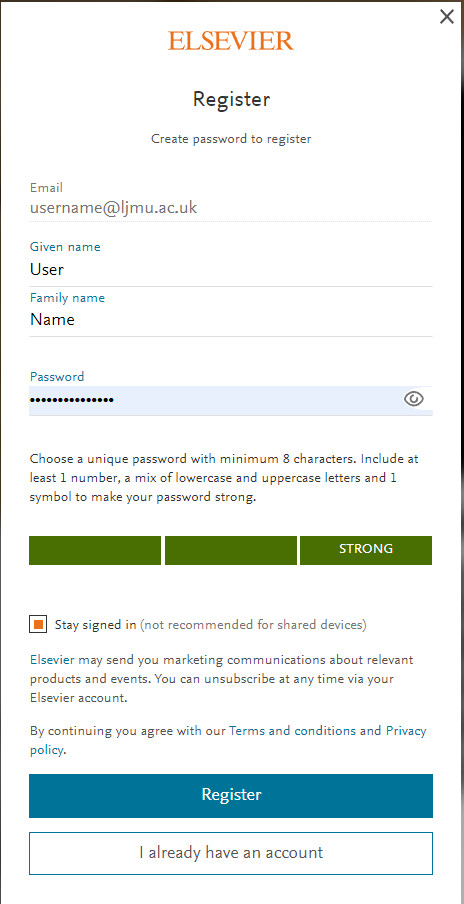


Click the Register button. You will be sent a confirmation email via the email address you have provided. Once confirmed, your account will be active. Please go to the dataset page using the link (by copy and paste the URL) we provided earlier. The page should look like the figure below:


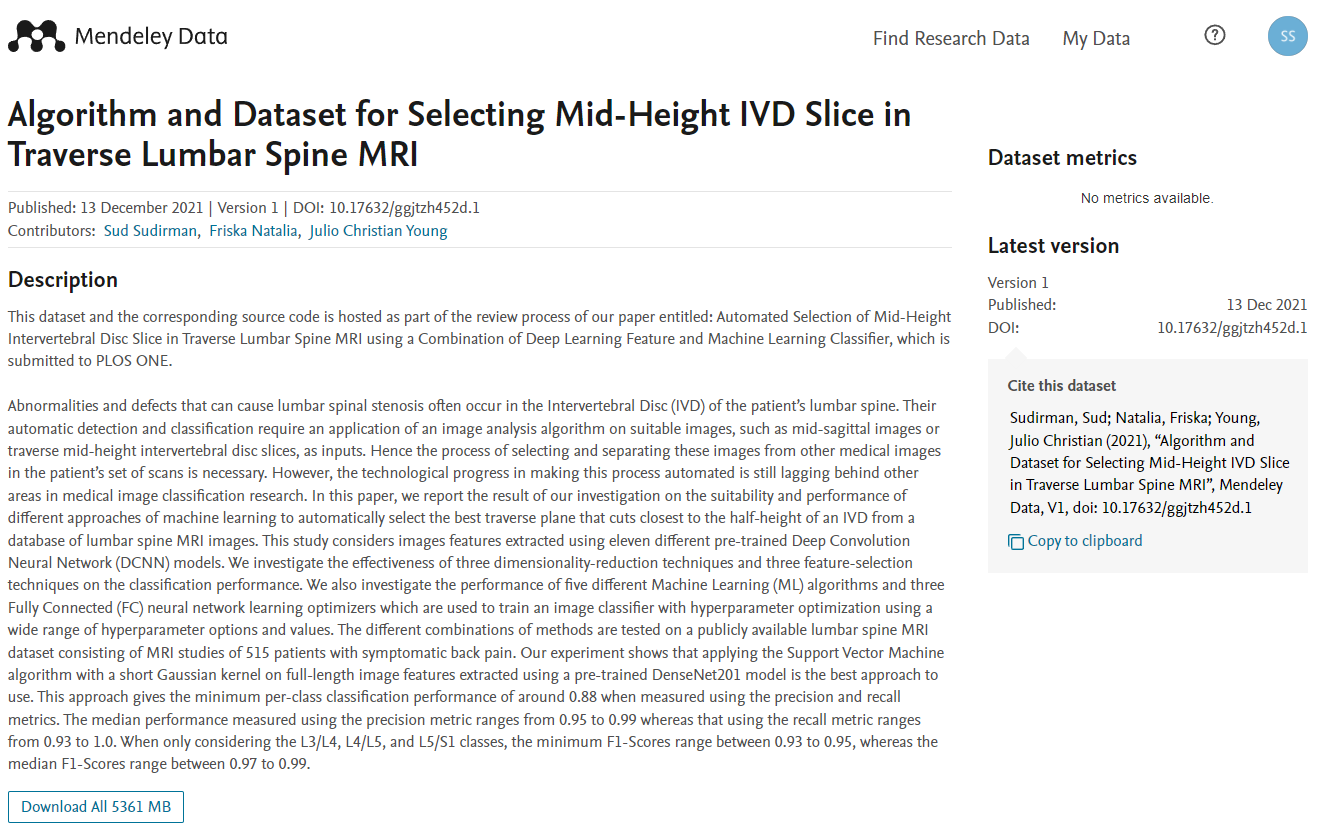


**Data Description**

After you download and unpack the Source and Dataset.zip file, you should see a directory structure as shown below. In this section, you can find information what each directory is and the files in it.

1. **\Source and Dataset\MRI Dataset\**


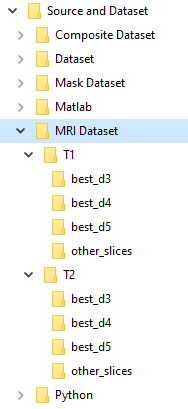


This folder contains 17,872 .ima files. These are DICOM files saved by the Siemens MRI scanners used in our research. There are T1 and T2 folders corresponding to the respective sequence type. The files in each folder are also categorised in different folders corresponding to the lumbar spine location.

515 files in T1 » best_d3 folder

515 files in T1 » best_d4 folder

515 files in T1 » best_d5 folder

7391 files in T1 » other_slices folder

515 files in T2 » best_d3 folder

515 files in T2 » best_d4 folder

515 files in T2 » best_d5 folder

7391 files in T2 » other_slices folder

Please refer to column 2 of Table 2 in the manuscript regarding these files.

Images in this folder are the ones used for the image registration process using **A00_ImageRegisterT1T2.mlx** in \Source and Dataset\Matlab\ folder.

1. **\Source and Dataset\Composite Dataset\**


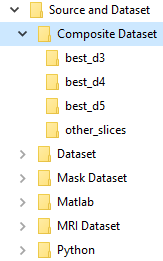


This folder contains 8,910 .png files. The files correspond to the MRI images after the registration process but before the Augmentation step.

513 files in best_d3 folder

513 files in best_d4 folder

513 files in best_d5 folder

7371 files in other_slices folder

Please refer to column 2 of Table 2 in the manuscript regarding these files.

Images in this folder are the ones used for the data Augmentation process using **A02_AugmentDataset.mlx** in \Source and Dataset\Matlab\ folder.

1. **Source and Dataset\Mask Dataset\**


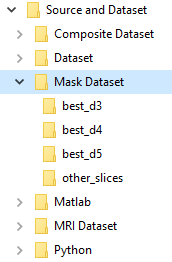


This folder contains 8,910 .png files. The files are by products of the image registration step. The files are black-and-white images with black pixels marking areas where T1 and T2 regions are properly aligned and white where they are not. So, we expect the majority of the files should be black images. Some do have small number of white pixels.

513 files in best_d3 folder

513 files in best_d4 folder

513 files in best_d5 folder

7371 files in other_slices folder

Images in this folder are not used in further experimentation but used to check the accuracy of the image registration process.

1. **\Source and Dataset\Dataset\**


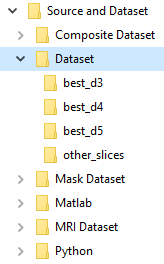


This folder contains 8,910 .png files. The files correspond to the MRI images after the registration process and the Augmentation step.

1026 files in best_d3 folder

1026 files in best_d4 folder

1026 files in best_d5 folder

1539 files in other_slices folder

Please refer to column 3 of Table 2 in the manuscript regarding these files.

Images in this folder are the ones used for model training and further experimentation.

1. **Source and Dataset\Matlab\**


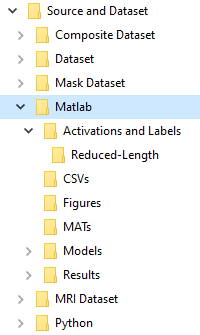


This folder contains the MATLAB implementation source code of the research. To reproduce the experiment results you can simply:

1. Run MATLAB

2. Run all *.MLX files in the alphabetical order

3. Python program is needed to dimensionality reduction using Factor Analysis / Fast ICA

3. The figures and tables in the paper can be reconstructed using:

a. X01_ShowOverallMetrics.mlx

b. X02_ShowSeparateMetrics.mlx

c. X03_ShowPerClassMetrics.mlx

Below is the description of what each .mls file does:

| **Step** | **MATLAB Program** | **Description** |
| --- | --- | --- |
| 1 | A00_ImageRegisterT1T2.mlx | This MATLAB program reads the .ima DICOM files in the subfolders of \Source and Dataset\MRI Dataset\T1 and \Source and Dataset\MRI Dataset\T2 folders and performs image registration on corresponding images and save the results in \Source and Dataset\Composite Dataset\ folder. The program also outputs a mask image for each pair, a black-and-white image with black pixels marking areas where T1 and T2 regions are properly aligned and white where they are not. These files are saved in a \Source and Dataset\Mask Dataset\ folder. |
| 2 | A01_ShowImageRegResults.mlx | This MATLAB Live Script reads a pair of T1-T2 MRI images and their registered composite image and display them individually or as a collage. |
| 3 | A02_AugmentDataset.mlx | This MATLAB Live Script augments the dataset in the \Source and Dataset\Composite Dataset\ folder by upsampling the number of images in the best_d3, best_d4, and best d5 folders (from 513 to 1026) and downsampling the number of images in the other_slices folder (7371 to 1039). |
| 4 | A03_SaveBottleneckFeatures.mlx | This MATLAB Live Script input the images to the pre-trained Deep Learning models and obtained the feature responses (the output of the last layer before the Classification Layer) and save them in \Activations and Labels folder. |
| 5 | A04_SaveFSModels.mlx | This MATLAB Live Script trains Feature Selection models using the full-length features and save the parameters in the Models folder. |
| 6 | A05_CalcFeatureLengths.mlx | This MATLAB Live Script constructs the shortFeatureLengths array that has the lengths of the reduced features for each combination of DCNN and FS/DR models. |
| 7 | C01_TrainML_FL.mlx | This MATLAB Live Script performs training of ML models using full-length features. |
| 8 | C02_TrainML_PCA.mlx | This MATLAB Live Script performs training of ML models using short length features reduced by PCA. |
| 9 | C03_TrainML_FastICA.mlx | This MATLAB Live Script performs training of ML models using short length features reduced by FastICA. |
| 10 | C04_TrainML_FA.mlx | This MATLAB Live Script performs training of ML models using short length features reduced by FA. |
| 11 | C05_TrainML_NCA.mlx | This MATLAB Live Script performs training of ML models using short length features reduced by NCA. |
| 12 | C06_TrainML_MRMR.mlx | This MATLAB Live Script performs training of ML models using short length features reduced by MRMR. |
| 13 | C07_TrainML_CHI2.mlx | This MATLAB Live Script performs This MATLAB Live Script performs training of ML models using short length features reduced by Chi-Square. |
| 14 | D01_TrainFCNN_FL.mlx |  |
| 15 | D02_TrainFCNN_PCA.mlx | This MATLAB Live Script performs This MATLAB Live Script performs training of Fully Connected Neural Network Classifiers using short length features reduced by PCA. |
| 16 | D03_TrainFCNN_FastICA.mlx | This MATLAB Live Script performs This MATLAB Live Script performs training of Fully Connected Neural Network Classifiers using short length features reduced by FastICA. |
| 17 | D04_TrainFCNN_FA.mlx | This MATLAB Live Script performs This MATLAB Live Script performs training of Fully Connected Neural Network Classifiers using short length features reduced by FA. |
| 18 | D05_TrainFCNN_NCA.mlx | This MATLAB Live Script performs This MATLAB Live Script performs training of Fully Connected Neural Network Classifiers using short length features reduced by NCA. |
| 19 | D06_TrainFCNN_MRMR.mlx | This MATLAB Live Script performs This MATLAB Live Script performs training of Fully Connected Neural Network Classifiers using short length features reduced by MRMR. |
| 20 | D07_TrainFCNN_CHI2.mlx | This MATLAB Live Script performs This MATLAB Live Script performs training of Fully Connected Neural Network Classifiers using short length features reduced by Chi-Square. |
| 21 | X00_CalculateMetrics.mlx | This MATLAB Live Script calculates the classification performance with using accuracy, precisio, recall, and fscore metrics. |
| 22 | X01_ShowOverallMetrics.mlx | This MATLAB Live Script displays the overall performance of the models. |
| 23 | X02_ShowSeparateMetrics.mlx | This MATLAB Live Script displays the models' performance in separate graphs. |
| 24 | X03_ShowPerClassMetrics.mlx | This MATLAB Live Script displays the per-class performance of the models. |

1. **Source and Dataset\Python\**

The Python folder contains the Python implementation of FA and FastICA, since the MATLAB’s implementation of the techniques unable to converge using our data. Instructions on how to compute the reduced features are given in Python Instruction to apply FA and FastICA.txt


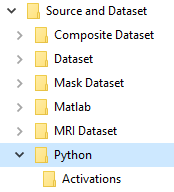

Supplement: S1 Data — Models and PYTHON/MATLAB source code to reproduce the results. (DOCX) [file pone.0261659.s001.docx]
