# Supplementary material for: Automated selection of mid-height intervertebral disc slice in traverse lumbar spine MRI using a combination of deep learning feature and machine learning classifier
Source: PLoS One. 2022 Jan 13;17(1):e0261659. doi: 10.1371/journal.pone.0261659 (PMC8758114; doi:10.1371/journal.pone.0261659)
Supplement: Attachment — Submitted filename: Response to Reviewers.docx [file pone.0261659.s003.docx]

Dear reviewers,

Thank you once again for all your effort and help in improving our paper. We appreciate your comments, suggestions, and critique. Based on your latest feedback, we have made the required changes to the paper. I hope that this latest version of the paper is of your satisfaction.

Kind regards

Sud

**Reviewer #1:**

1) "A CAD system can help doctors understand the cause of an illness better by automating some steps in the diagnosis process. In a CAD system that uses medical images, the system applies image analysis algorithms to different types or modalities of medical imaging, such as Magnetic Resonance Imaging (MRI), of the patient."- add reference for this statement. I suggest the following:

- Skin lesion segmentation and multiclass classification using deep learning features and improved moth flame optimization

- Computer Decision Support System for Skin Cancer Localization and Classification

- Multimodal brain tumor classification using deep learning and robust feature selection: A machine learning application for radiologists

*We have added the relevant references to the paper. That sentence now reads, “A CAD system can help doctors understand the cause of an illness better by automating some steps in the diagnosis process. In a CAD system that uses medical images, the system applies image analysis algorithms to different types or modalities of medical imaging, such as Magnetic Resonance Imaging (MRI), of the patient [1–3].”*

*The added references are:*

*1.	Khan MA, Sharif M, Akram T, Damaševičius R, Maskeliūnas R. Skin lesion segmentation and multiclass classification using deep learning features and improved moth flame optimization. Diagnostics. 2021;11(5):811.*

*2.	Khan MA, Akram T, Sharif M, Kadry S, Nam Y. Computer Decision Support System for Skin Cancer Localization and Classification. C Mater Contin. 2021;68(1):1041–64.*

*3.	Khan MA, Ashraf I, Alhaisoni M, Damaševičius R, Scherer R, Rehman A, Bukhari SAC. Multimodal brain tumor classification using deep learning and robust feature selection: A machine learning application for radiologists. Diagnostics. 2020;10(8):565.*

2) "In the case of MRI, for example, a CAD system might use the two modalities of MRI, namely the T1-weighted and T2-weighted MRI, which can differently highlight various types of tissues based on their fat and water composition."- add figures of T1, T2, T1W, and Flair. You can take this figure from the following:

- A Decision Support System for Multimodal Brain Tumor Classification using Deep Learning

- Microscopic brain tumor detection and classification using 3D CNN and feature selection architecture

*Unfortunately, the figures in those papers are copyrighted materials and we are not allowed to reproduce them in our paper without the publisher’s permission. In their place, we added two examples taken from our dataset. Since we only used T1- and T2-weighted images hence it is only appropriate that we include these two examples only. In the paper, after that sentence we insert the following sentence: “An example of a T1-weighted and a T2-weighted traverse MRI images of the L3/L4 Intervertebral Disc (IVD) of the same patient are shown in Figure 1.”*

*Figure 1. A T1-weighted (left) and a T2-weighted (right) traverse MRI images of the L3/L4 Intervertebral Disc of a patient are shown. One marked difference in the two images is the cerebrospinal fluid (CSF) in the spinal canal that appears black on the T1-weighted image but as a brighter region on the T2-weighted image because of its low fat contents.*

*I hope this additional information is a sufficient substitute to your original suggestion.*

3) "Training DCNN models take a long time hence there exist several pre-trained DCNN models that are readily usable for image classification."- add reference for this statement.

*We have added the relevant references to the paper. That sentence now reads, “Training DCNN models take a long time hence there exist several pre-trained DCNN models that are readily usable for image classification [29,30]”*

*The added references are:*

*29. Kornblith S, Shlens J, Le Q V. Do better imagenet models transfer better? In: Proceedings of the IEEE/CVF Conference on Computer Vision and Pattern Recognition. 2019. p. 2661–71.*

*30. Morid MA, Borjali A, Del Fiol G. A scoping review of transfer learning research on medical image analysis using ImageNet. Comput Biol Med. 2020;128:104115.*

4) "used in many other types of image classification tasks, including medical image classification, through a method called Transfer Learning"- add reference for this statement: I suggest the folowing:

- Attributes based skin lesion detection and recognition: A mask RCNN and transfer learning-based deep learning framework

- A deep neural network and classical features based scheme for objects recognition: an application for machine inspection

*We have added the relevant references to the paper. The complete sentence now reads, “However, despite being extracted using a model trained using photographic images, these learnable features are sufficiently general that they can be used in many other types of image classification tasks, including medical image classification, through a method called Transfer Learning [32,33], which process is elucidated in Figure 4.”*

*The added references are:*

*32.	Khan MA, Akram T, Zhang Y-D, Sharif M. Attributes based skin lesion detection and recognition: A mask RCNN and transfer learning-based deep learning framework. Pattern Recognit Lett. 2021;143:58–66.*

*33.	Hussain N, Khan MA, Sharif M, Khan SA, Albesher AA, Saba T, Armaghan A. A deep neural network and classical features based scheme for objects recognition: an application for machine inspection. Multimed Tools Appl. 2020;1–23.*

5) Add manuscript organization before materials and methods section.

*We have added paper organization before materials and methods section that reads: “The organization of this paper is as follows. Section 2 describes the dataset used in the research and the proposed method. The experimental results, analysis, and discussion are discussed in detail in Section 3. We then provide the conclusion of our findings in the last section of the paper.”*

6) What represent Table 1?

*Table 1 shows the range of acquisition parameter values used during traverse MRI scan. The acquisition parameters are a set of values that the radiologist or technician used when the MRI images are being recorded. The values can be fixed (e.g., in case of Field of View, Matrix, Imaging Frequency, and Flip Angle) or differ from one patient to another depending on the decision made by the technician at that time. This information is extracted from the DICOM images from the dataset and description of each parameter can be found on https://dicom.innolitics.com/ciods/ct-performed-procedure-protocol/performed-ct-reconstruction/00189934/00180050*

*We have altered the caption of the table slightly to improve the description of the table. It now reads,*

*Table 1. The range of acquisition parameter values used during traverse MRI scans*

*Also, for your information, Table 1 is included in the paper because in the previous round of review one of the reviewers required us to include the technical detail of the dataset.*

*Thank you for your feedback. I hope al the changes that we made above have addressed all your concerns.*

**Reviewer #2:** The revision of opinions is not satisfactory. The overall expression and organization of the paper should be further improved.

*Thank you for your feedback. I hope the changes that we made as part of the review from the other reviewer have addressed all your concerns.*

**Reviewer #3:** (No Response)
