# Supplementary material for: Automated selection of mid-height intervertebral disc slice in traverse lumbar spine MRI using a combination of deep learning feature and machine learning classifier
Source: PLoS One. 2022 Jan 13;17(1):e0261659. doi: 10.1371/journal.pone.0261659 (PMC8758114; doi:10.1371/journal.pone.0261659)
Supplement: Attachment — Submitted filename: Response to Reviewers.docx [file pone.0261659.s004.docx]

Dear editors and reviewers,

Thank you once again for all your effort and help in improving our paper. We appreciate your comments, suggestions, and critique. Based on your latest feedback, we have made the required changes to the paper but also at the same time will attempt to make some clarification on some points that may have been missed.

Kind regards

Sud

**Reviewer #2:**

1. If the authors have adequately addressed your comments raised in a previous round of review and you feel that this manuscript is now acceptable for publication, you may indicate that here to bypass the “Comments to the Author” section, enter your conflict of interest statement in the “Confidential to Editor” section, and submit your "Accept" recommendation.

Reviewer #2: All comments have been addressed

2. Is the manuscript technically sound, and do the data support the conclusions?

Reviewer #2: Partly

3. Has the statistical analysis been performed appropriately and rigorously?

Reviewer #2: No

*We believe we have included comprehensively the experimental results in the manuscript. We implemented each of the 759 method combinations 20 times, each with a different combination of training and test sets, to provide us with statistically representative results. And we shown the average result of the 20 repeats of each method combination using four performance metrics.*

4. Have the authors made all data underlying the findings in their manuscript fully available?

The PLOS Data policy requires authors to make all data underlying the findings described in their manuscript fully available without restriction, with rare exception (please refer to the Data Availability Statement in the manuscript PDF file). The data should be provided as part of the manuscript or its supporting information, or deposited to a public repository. For example, in addition to summary statistics, the data points behind means, medians and variance measures should be available. If there are restrictions on publicly sharing data—e.g. participant privacy or use of data from a third party—those must be specified.

Reviewer #2: No

*We had made our raw MRI data, PNG images, MATLAB and PYTHON source code, and experimental results available in all previous submissions through Mendeley Data (link provided in the Data Statement). This section, if we recall correctly, has not been commented as NO before. We believe that there may be a confusion on how to download the file since in order to access the file one has to have an Elsevier account. Creating this account is free. So, we added more information on the Data Statement document regarding this.*

5. Is the manuscript presented in an intelligible fashion and written in standard English?

PLOS ONE does not copyedit accepted manuscripts, so the language in submitted articles must be clear, correct, and unambiguous. Any typographical or grammatical errors should be corrected at revision, so please note any specific errors here.

Reviewer #2: Yes

6. Review Comments to the Author

Please use the space provided to explain your answers to the questions above. You may also include additional comments for the author, including concerns about dual publication, research ethics, or publication ethics. (Please upload your review as an attachment if it exceeds 20,000 characters)

Reviewer #2:

1. A large number of deep learning models have been applied to experiments, but their training details have been ignored.

*The training details of each model were given in Table 4 and Table 5. The tables contain both the fixed settings (the ones that do not get searched by the hyperparameter optimization process) and the variable settings/parameters (the ones that are searched automatically by the hyperparameter optimization process).*

*But we have rechecked the information in the table for completeness to make sure we have included all the training parameters and settings. As a result, we have added three additional parameters to Table 5 (for Fully Connected Neural Network training).*

2. Many figures are still of low quality and cannot be seen clearly.

*We have made queries to PLOS ONE regarding this since we have followed all the required steps to produce the figures. The original figures that we uploaded are of highest quality and very clear but for some reason when they are embedded to the pdf their quality is reduced significantly. However, the original image file can be accessed by clicking the link at the top-right of the page where the figure is displayed (see the illustration below).*

3. Intermediate experimental results should also be presented.

*Unfortunately, we cannot make this change since we are not sure what intermediate experimental results are needed. We have tried searching the literature for what intermediate experimental results might be, but we cannot find anything. We have also reached out to the PLOS ONE editorial team requesting for more description from the reviewers. Unfortunately, the team did not receive any response from the reviewers by the resubmission deadline. However, it is our we firm belief that our paper has included all the necessary results to base our findings. Furthermore, since we made the data and source code available, the readers should be able to reproduce the experimental results including any intermediate results if so desired.*
